# Intracellular Cholesterol Synthesis and Transport

**DOI:** 10.3389/fcell.2022.819281

**Published:** 2022-03-21

**Authors:** Qingyang Shi, Jiahuan Chen, Xiaodong Zou, Xiaochun Tang

**Affiliations:** ^1^ Center of Reproductive Medicine and Center of Prenatal Diagnosis, The First Hospital, Jilin University, Changchun, China; ^2^ Jilin Provincial Key Laboratory of Animal Embryo Engineering, College of Animal Sciences, Jilin University, Changchun, China; ^3^ Chongqing Research Institute of Jilin University, Chongqing, China

**Keywords:** cholesterol synthesis, cholesterol trafficking, intracellular, endoplasmic reticulum, sterol regulatory element-binding proteins

## Abstract

Cholesterol homeostasis is related to multiple diseases in humans, including cardiovascular disease, cancer, and neurodegenerative and hepatic diseases. The cholesterol levels in cells are balanced dynamically by uptake, biosynthesis, transport, distribution, esterification, and export. In this review, we focus on *de novo* cholesterol synthesis, cholesterol synthesis regulation, and intracellular cholesterol trafficking. In addition, the progression of lipid transfer proteins (LTPs) at multiple contact sites between organelles is considered.

## Introduction

The endoplasmic reticulum (ER) produces a number of phospholipids and sterols as well as triglycerides, cholesterol esters, and ceramide. Lipids are exported from the ER to the plasma membrane (PM) and other organelles that lack the ability to synthesize lipids on their own. Despite the extensive transport of lipids and other materials between organelles, these structures exhibit remarkable differences in lipid composition and quantity (Holthuis and Menon, 2014). Cholesterol is an essential component of PMs; it couples with sphingolipid and glycosylphosphatidylinositol (GPI)-anchored proteins to form dynamic and nanoscale domains that are distributed in both inner and outer leaflets of the cell membrane (Lingwood and Simons, 2010; Raghupathy et al., 2015) and participate in the regulation of cellular processes. The proportion of cholesterol compared to that of all lipids in the ER is only 5 mol% but reaches 30–40 mol% in the PM (Van Meer et al., 2008). In this review, we discuss how cholesterol is synthesized in the ER and how the cholesterol is transferred between organelles, especially LTPs, which responsible for bulk transport of cholesterol, and are involved.

### Cholesterol Synthesis

**FIGURE 1:**
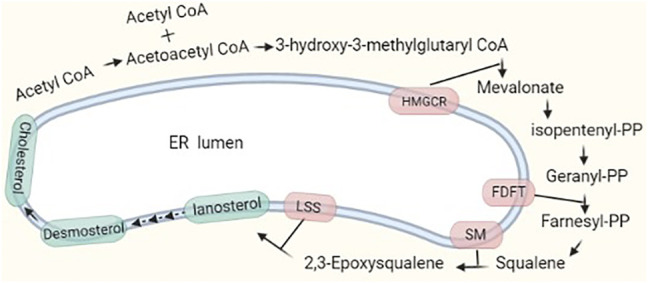
Major pathway of cholesterol synthesis in cells. Two molecules of acetyl-CoA condense and form HMG-CoA with the addition of a third acetyl-CoA molecule. The process to form cholesterol involves nearly 30 reaction steps. There are two rate-limiting steps catalyzed by HMGCR and SM.

Cholesterol levels in cells are regulated dynamically by *de novo* biosynthesis, exogenous uptake, storage, and exportation. Approximately 700–900 mg of cholesterol per day is produced through *de novo* synthesis in humans, while 300–500 mg is taken up from the diet. Approximately 50% of the total synthesized cholesterol comes from the liver. Endogenous and exogenous cholesterol are metabolized into bile acids at approximately 400 mg/day and into steroid hormones at approximately 50 mg/day; the rest is excreted in feces and by the skin (Russell, 1992). The process of cholesterol synthesis is described in the following. In brief, two molecules of acetyl-coenzyme A (CoA) form acetoacetyl-CoA, and the addition of a third molecule to form 3-hydroxy-3-methylglutaryl CoA (HMG-CoA) is catalyzed by HMG-CoA synthase. HMG-CoA is reduced to mevalonate by HMG-CoA reductase, and this reaction is highly regulated at the transcriptional and posttranslational levels by metabolic intermediates. Mevalonate undergoes extensive phosphorylation and decarboxylation to form isopentenyl pyrophosphate (IPP), and IPP continues to be polymerized to form farnesyl pyrophosphate (FPP) (Figure 1). FPP formation is followed by one of three key steps: condensation of two FPP molecules to form squalene, which is processed to cholesterol; combination of a series of condensed IPPs with one molecule of FPP to form a long *trans* polyprenyl derivative, which is a side chain of ubiquinone, and sequential addition of IPP to FPP to form dolichols (Rudney and Sexton, 1986; Russell, 1992). Cholesterol synthesis is completed in the ER membrane, and cholesterol homeostasis is tightly regulated. We briefly review the key players in the regulation of *de novo* cholesterol synthesis.

### Regulation of Cholesterol Synthesis

Cholesterol levels are regulated dynamically, and there are three key factors in cholesterol synthesis: sterol regulatory element-binding protein 2 (SREBP2), 3-hydroxy-3-methylglutaryl coenzyme A reductase (HMGCR), and squalene monooxygenase (SM). The regulation occurs in three dimensions: transcriptional, translational, and posttranslational (Table 1).

**TABLE 1:** Molecules implicated in SREBP2, HMGCR, and SM regulation.

Regulation	SREBP2	HMGCR	SM
Transcription	Sp1, NF-Y, nSREBP2, FoxO3, SIRT6, SIRT1, MARCH6	SREBP1, nSREBP2, NF-kB, c-Fos, c-Jun, HSP-70, HO-1	NF-1, Sp1, YY1, c-Myc, IRF-1, Mir-133b
Post-translation	Erlins, RNF145, RNF5, TRC8, PCK1, Brg1, POST1, Fbw7, SIRT1, p300, CBP	Gp78, TRC8, RNF145, USP20, UBIAD1	MARCH6, UBE2G2, UBE2J2, Squalene

#### Sterol Regulatory Element-Binding Protein 2

SREBP2 is an isoform of the SREBP transcription factor family and a master regulator of lipid homeostasis that specializes in cholesterol synthesis (Brown and Goldstein, 1997). SREBP2 is synthesized as an inactive precursor that binds to the ER membrane and is composed of three domains: an N-terminal transactivation domain for DNA binding and dimerization; a hydrophobic transmembrane domain separated by a lumen facing, 30 aa short loop; and a C-terminal regulatory domain responsible for interaction with SREBP cleavage activating protein (SCAP) (Goldstein et al., 2006). To become an active form from a precursor, SREBP2 translocates to the Golgi apparatus from the ER. In the Golgi apparatus, there are two proteases, the site 1 and site 2 proteases (S1P and S2P), that cleave the SREBP2 precursor sequentially to liberate the N-terminus, which enter the nucleus as a homodimer to bind sterol regulatory element (SRE) sequences in the promoter to activate target genes (Brown et al., 2018).

At the transcriptional level, the SREBP2 gene promoter has a 10-bp SRE, 6-bp Sp1 (Sp1 transcription factor), and NF-Y (nuclear transcription factor Y) binding sites, which coincide with other SREBP2-targeted cholesterol synthesis genes. The existence of an SRE binding site means that it is regulated by its activated form nSREBP2 (Sato et al., 1996). SREBP2 transcription is also epigenetically regulated (Tao et al., 2013). There is a FoxO3 (forkhead box O3) binding sequence and insulin response element (IRE) in the promoter. FoxO3 recruits Sirt6 (sirtuin 6), and Sirt6 deacetylates histone H3 and inhibits SREBP2 transcription.

**FIGURE 2:**
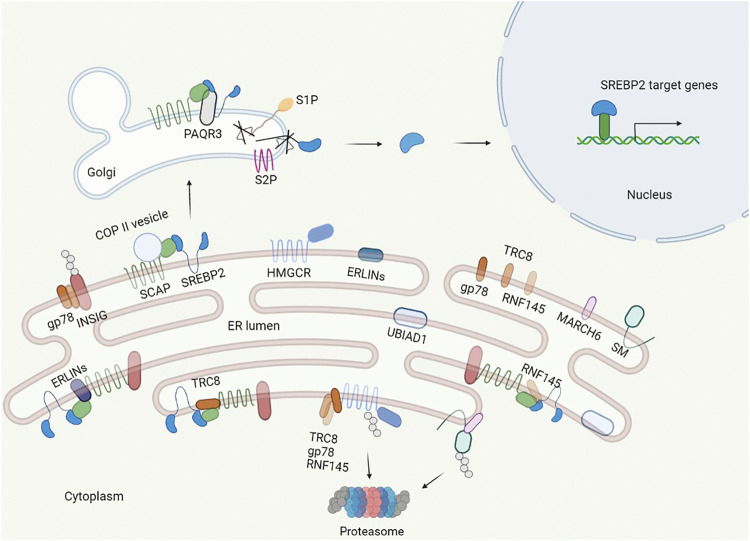
Regulation of cholesterol synthesis. The central transcription factor of cholesterol synthesis in cells is SREBP2, which is regulated in multiple layers and controls the synthesis of key enzymes in cholesterol synthesis. SREBP2 binds to SCAP in the ER. When the cholesterol level is less than 5% of the ER, SCAP binds to the COPII protein and escorts SREBP2 from the ER to the Golgi and anchors *via* adipoQ receptor 3 (PAQR3) in the Golgi, where site 1 and site 2 proteases (S1P, S2P) cleave the luminal loop of SREBP2 to release the N-terminal domain that enters the nucleus. In the nucleus, SREBP2 activates multiple cholesterol synthesis genes by binding to the SRE. Additionally, the INSIG proteins dissociate from SCAP, and HMGCR and SM are less bound and ubiquitylated by E3 ubiquitin ligase and degraded by the proteasome. When the cholesterol level is more than 5% of the ER, the INSIGs are recruited by SCAP to form the SCAP–SREBP2–INSIG complex, and the complex holds in the ER further by ERLINs and TRC8. Additionally, cholesterol induces the E3 ligase complex to ubiquitylate HMGCR and SM.

The process of maturation of SREBP2 is triggered by the cholesterol concentration in the ER (Figure 2). When the ER cholesterol level is below 5 mol% of all ER lipids, SCAP, an escort of SREBP2, exerts conformational changes to dissociate from insulin-induced gene (Insig)-1, which is complexed with SCAP-SREBP2 in sterol abundance, and binds to COPII coat proteins to transport SREBP2 to the Golgi apparatus *via* vesicles (Nohturfft et al., 2000; Shimano and Sato, 2017). If Insig-1 levels are elevated, the cholesterol concentration that triggers SCAP transport of SREBP2 is lowered to 3 mol% (Radhakrishnan et al., 2008, 2009). After separation with the SCAP-SREBP2 complex under depletion of cholesterol, Insig-1 is ubiquitinated and degraded with a half-life within 30 min (Goldstein et al., 2006). Nuclear SREBP2 targets corresponding genes, including Insig-1, and newly synthesized Insig-1 is degraded continually until the cholesterol level is above 5 mol% in the ER. Stabilized Insig-1 halts the SCAP-SREBP2 complex in the ER membrane, inhibits SREBP2 maturation, and stops cholesterol synthesis. Again, if the ER cholesterol level is below 5 mol% of all ER lipids, feedback regulation is triggered. Insig-2 has similar functions to Insig-1, but it is expressed constitutively at low levels and is not regulated by SREBP2 (Goldstein et al., 2006).

Additional proteins participate in the process of SREBP2 maturation in the ER and Golgi apparatus. Erlins localized to the ER lumen with heteromultimeric complexes are cholesterol-binding proteins that interact with Insig-SCAP-SREBP2 to restrict SREBP2 activation in the presence of cholesterol (Huber et al., 2013). RNF145 and RNF5 are ER-anchored E3 ubiquitin ligases; RNF145 ubiquitinates lysine residues of SCAP (K454, K466) and damages binding with COPII protein and Golgi transport (Jiang et al., 2018), and RNF5 binds to the transmembrane domain and ubiquitinates SCAP at K305, regulating the process of SREBP2 maturation (Kuan et al., 2020). TRC8 is also a ubiquitin ligase that binds to the SCAP-SREBP2 complex, and the TRC8-SCAP-SREBP2 complex hinders the interaction of SCAP with COPII proteins independent of its ubiquitin ligase activity (Irisawa et al., 2009). Phosphoenolpyruvate carboxykinase 1 (PCK1), a rate-limiting enzyme in gluconeogenesis, is phosphorylated by AKT in hepatocellular carcinoma (HCC) patients, and phosphorylated PCK1 translocates to the ER and acts as protein kinase to phosphorylates Insig-1 and Insig-2 in the ER (Xu et al., 2020). Phosphorylated Insigs have weak binding ability with sterols and disrupt the SCAP-SREBP2 complex, promoting the translocation of SCAP-SREBP2 to the Golgi from the ER. Although the relationship between phosphorylated PCK1 mediating SREBP2 activation and sterol levels is unknown, more attention needs to be paid to this relationship, especially in nonphysiological conditions. In 2021, Brahma-related gene 1 (Brg1) and partner of site-1 protease (POST1) were identified as cofactors involved in SREBP2 regulation. Brg1, a chromatin remodeling protein, interacts with Sp1 at the promoter of SCAP, activates the transcription of SCAP, and promotes the maturation of SREBP2 (Kong et al., 2021). On the other hand, Brg1 is recruited to the promoters of cholesterogenic genes by SREBP2. In turn, Brg1 recruits the H3K9 methyltransferase KDM3A to promote the transcription of related genes, and deficiency of Brg1 in the liver reduces cholesterol levels in mice (Fan et al., 2020). POST1 was discovered by a genome-wide CRISPR/Cas9 knockout screen; it controls S1P maturation, and ablation of POST1 decreases nuclear SREBP2 and the corresponding target gene expression (Xiao et al., 2021).

After maturation, the activated form of nSREBP2 can be phosphorylated by GSK-3 at T426 and S430, the surrounding sequence called phosphodegron. Fbw7, which is a substrate receptor of SCF ubiquitin ligase, interacted with the phosphodegron to degrade it and decrease the expression of SREBP2 target genes (Sundqvist et al., 2005). Simultaneously, p300 and CBP acetylate, the N-terminus of SREBP2, is stabilized and enhances the expression of SREBP2 target genes (Giandomenico et al., 2003). Accordingly, the acetylation of SREBP2 by SIRT1 inhibition also increases SREBP2 stability and transcriptional activity. Moreover, SREBP2 can be phosphorylated by ERK, MAPK, AMPK, and mTORC1 and SUMOylated to regulate stability, transcriptional activity, and trafficking (Arito et al., 2008; Mohamed et al., 2018; Liu et al., 2020).

##### 3-Hydroxy-3-Methylglutaryl Coenzyme A Reductase

HMGCR is a membrane protein located in the ER that contains a transmembrane domain in the N-terminus and a hydrophilic C-terminus facing the cytosol. The cytosol-facing C-terminal domain is responsible for mevalonate formation, and the transmembrane domain includes an SSD, like SCAP, that senses sterol levels in the ER. *HMGCR* transcription is stimulated by nSREBP2, similar to other cholesterol-related genes. To date, HMGCR posttranslational regulation, including ubiquitination and phosphorylation, has been extensively studied.

HMGCR can be ubiquitylated by the intermediates of mevalonate and cholesterol derivatives, such as lanosterol, 24, 25-dihydrolanosterol, and 25- and 27-hydroxycholesterol (Song et al., 2005; Chen et al., 2019). However, cholesterol itself has a minor degradation effect. In addition to the cholesterol pathway, *γ*- and *δ*-tocotrienols also mimic sterols but not nonsterol isoprenoids, promoting the degradation of HMGCR (Song and DeBose-Boyd, 2006).

HMGCR ubiquitination and degradation induced by sterol requires Insig binding. The second transmembrane helix of HMGCR contains a YIYF sequence, which also exists in the SSD of SCAP and is responsible for Insig binding and ubiquitination. Mutation of tetrapeptide YIYF abolishes Insig binding of HMGCR, as in SCAP, and destroys sterol-induced endoplasmic reticulum-associated degradation (ERAD) (Jiang et al., 2018). Substitution of lysine 248 with arginine in HMGCR abolishes ubiquitination and delays degradation but does not affect Insig binding (Sever et al., 2003). Another substitution at lysine 89 further delays degradation in the absence of lysine 248 but has little effect on its own. Lysine 248 is near the C-terminal catalytic domain and localizes to the juxtamembrane; this position may facilitate ubiquitin transfer to HMGCR by membrane-bound ubiquitin transferase and subsequent ERAD.

The ubiquitination of HMGCR is mediated by membrane-bound E3 ubiquitin ligase. At least three E3 ligases have been reported to be related to Insigs. Gp78, known as AMFR, is an ER membrane-anchored ubiquitin ligase that mediates HMGCR ubiquitination by interacting with Insigs. When cholesterol is abundant, gp78 is transferred to the HMGCR-Insig complex, causing the ubiquitination of HMGCR and degradation and suppressing cholesterol synthesis; when cholesterol is depleted, HMGCR is free from the Insig-gp78 complex and stabilized, increasing cholesterol synthesis (Jo et al., 2011b; Liu et al., 2012). Another ubiquitin ligase is TRC8, known as RNF139. Insig binding to HMGCR recruits Trc8 to facilitate its ubiquitination. Either gp78 or TRC8 knockdown in cells inhibits sterol-induced degradation by approximately 50%–60%, and combined knockdown of the two E3 ubiquitin ligases inhibits the degradation of HMGCR by up to 90% (Jo et al., 2011a). RNF145 is an E3 ubiquitin ligase that interacts with Insigs to ubiquitinate HMGCR. Knockdown of both RNF145 and gp78 abrogates HMGCR degradation, but RNF145 itself has little effect on HMGCR stability (Menzies et al., 2018). RNF145 also contains a YIYF sequence in the SSD, which is essential for its binding with Insigs, and in the RING finger domain, the Cys537 residue is responsible for RNF145 activity (Jiang et al., 2018). Why multiple E3 ubiquitin ligases are involved in HMGCR degradation and which ligases are responsible for HMGCR ubiquitination under certain conditions need further investigation. In contrast to ubiquitination, HMGCR is deubiquitinated by mTORC1-phosphorylated USP20, which preferentially hydrolyzes K48 and K63 linkages, and stabilized HMGCR increases cholesterol synthesis in the feeding state (Lu et al., 2020).

After ubiquitination of HMGCR, energy produced from VCP/p97-mediated hydrolysis of ATP powers ubiquitinated HMGCR extraction from the ER membrane (Stevenson et al., 2016). The extracted HMGCR is transferred to the cytosol from the ER membrane by the 19S regulatory subunit of the proteasome. Subsequently, it is delivered to the proteolytic core of the 20S proteasome for degradation. Both VCP and the 19S regulatory subunit have AAA + ATPase activity (Meyer et al., 2012). The extraction process is enhanced by geranylgeraniol, which is a derivative of isoprenoid geranylgeranyl pyrophosphate (GGpp). In the presence of a substrate of GGpp, UBIAD1, which binds with HMGCR and blocks its membrane extraction, is transported to the Golgi and removes the inhibition of HMGCR degradation. UBIAD1 is a membrane prenyltransferase that can catalyze the transfer of isoprenyl groups to aromatic acceptors and produce ubiquinones, hemes, chlorophylls, vitamin E, and vitamin K. UBIAD1 knockout in mice is embryonic lethal, and the phenotype can be rescued by knocking in HMGCR, which is a resistant mutant (Schumacher et al., 2015; Jo et al., 2020). Therefore, HMGCR levels can be regulated with nonsterol mevalonate pathway products.

Another posttranslational regulation of HMGCR is phosphorylation. The Ser872 residue in the C-terminal catalytic domain of HMGCR is phosphorylated by AMPK, and phosphorylation at Ser872 disrupts HMGCR activity and restricts the flux of the mevalonate pathway rapidly but does not affect sterol-induced ubiquitination and subsequent degradation (Clarke and Hardie, 1990).

#### Squalene Monooxygenase

SM catalyzes the first oxygenation step in cholesterol synthesis; it introduces an epoxide group to squalene, converts alkene squalene into squalene epoxide, and is proposed to be a rate-limiting step in cholesterol synthesis.

There are three SREs in the SM promoter: two adjacent SREs near the initiation site that partially respond to sterol via SREBP2 and a third SRE that is sterol independent. Other transcriptional cofactors and factors, including NF-Y, Sp1, YY1, c-Myc, and IRF-1, participate in the regulation of SM transcription (Chua et al., 2020). Mir-133b is reported to promote SM mRNA degradation (Qin et al., 2017).

The focus of SM regulation, similar to that of HMGCR, is posttranslational. SM can be ubiquitinated and degraded under cholesterol abundance. The phenomenon of cholesterol-induced squalene accumulation suggests that SM, similar to HMGCR, is another flux-controlling enzyme (Gill et al., 2011).

The N-terminal 100 residues of SM contain a cholesterol-sensitive amphipathic helix and a reentrant loop; the amphipathic helix binds membranes with absent cholesterol, the affinity is reduced upon cholesterol addition, and the released helix forms a disordered sequence (Chua et al., 2017; Prinz, 2017). MARCH6, an E3 ligase that physically interacts with conformationally changed SM (Zelcer et al., 2014), combines with two E2 enzymes, UBE2G2 and UBE2J2, for ubiquitination and subsequent degradation in the presence of cholesterol. In contrast to cholesterol-induced SM degradation, the accumulated substrate squalene binds to the N-terminal 100 residues of SM, altering the recognition of MARCH6, and stabilizing SM on the ER membrane (Yoshioka et al., 2020). In addition to ubiquitination, MARCH6 can regulate SREBP2 at the transcriptional level; thus, HMGCR and SM are controlled. During ERAD, SM is truncated by N-terminal degradation, which results in defects in sterol sensing. Truncated SM has similar abundance and is constitutively active. The distinction of SM and truncated SM function needs further investigation in detail.

### Intracellular Cholesterol Transport

Cholesterol is distributed unevenly in cellular membranes. The PM is the membrane most enriched with cholesterol and accounts for approximately 30–40 mol% of total cholesterol in cells; cholesterol is also abundant in the endocytic recycling compartment and *trans*-Golgi facing side (Lange, 1991; Pomorski et al., 2001; Ikonen, 2008). The ER, mitochondria, and lysosomes are characterized by small amounts of cholesterol (Maxfield and Wüstner, 2002). To achieve compositional heterogeneity, cholesterol needs to be transported in cells in a dedicated manner.

The synthesized cholesterol in the ER is transported to organelles immediately, and this cholesterol transport is primarily coupled with the transport and metabolism of phosphoinositide, phosphatidylserine (PtdSer), and sphingolipids (Holthuis and Menon, 2014). Cholesterol trafficking is mediated by vesicular and nonvesicular trafficking systems (Prinz, 2010; Luo et al., 2019). Vesicular transport plays an important role in the response to trafficking of proteins in extracellular and endocytic pathways, and along with protein transport, cholesterol can traffic between organelles in the secretory pathway continuously (Holthuis and Menon, 2014). However, a number of lines of evidence support that there is an alternative nonvesicular transport response for rapid and bulk cholesterol exchanges in the secretory pathway that do not receive vesicular trafficking.

The nonvesicular transport system includes cholesterol traveling spontaneously between membranes at a low rate of desorption and movement, horizontal movement in continuous membranes, and movement in two leaflets of the membranes. *In vitro* investigations have demonstrated that the spontaneous exchange of cholesterol is related to aqueous-phase solubility and membrane curvature. Cholesterol exchanges rapidly from donors of small vesicles that have higher membrane curvature than large vesicles (Lev, 2010). However, cholesterol interacts with sphingolipid and GPI-anchored proteins to form condensed complexes in the bilayer, and the nanostructure decreases the desorption of cholesterol from membranes. Lipid transfer proteins (LTPs) have been identified to accelerate the transport of lipids, including cholesterol (Wong et al., 2019).

**FIGURE 3:**
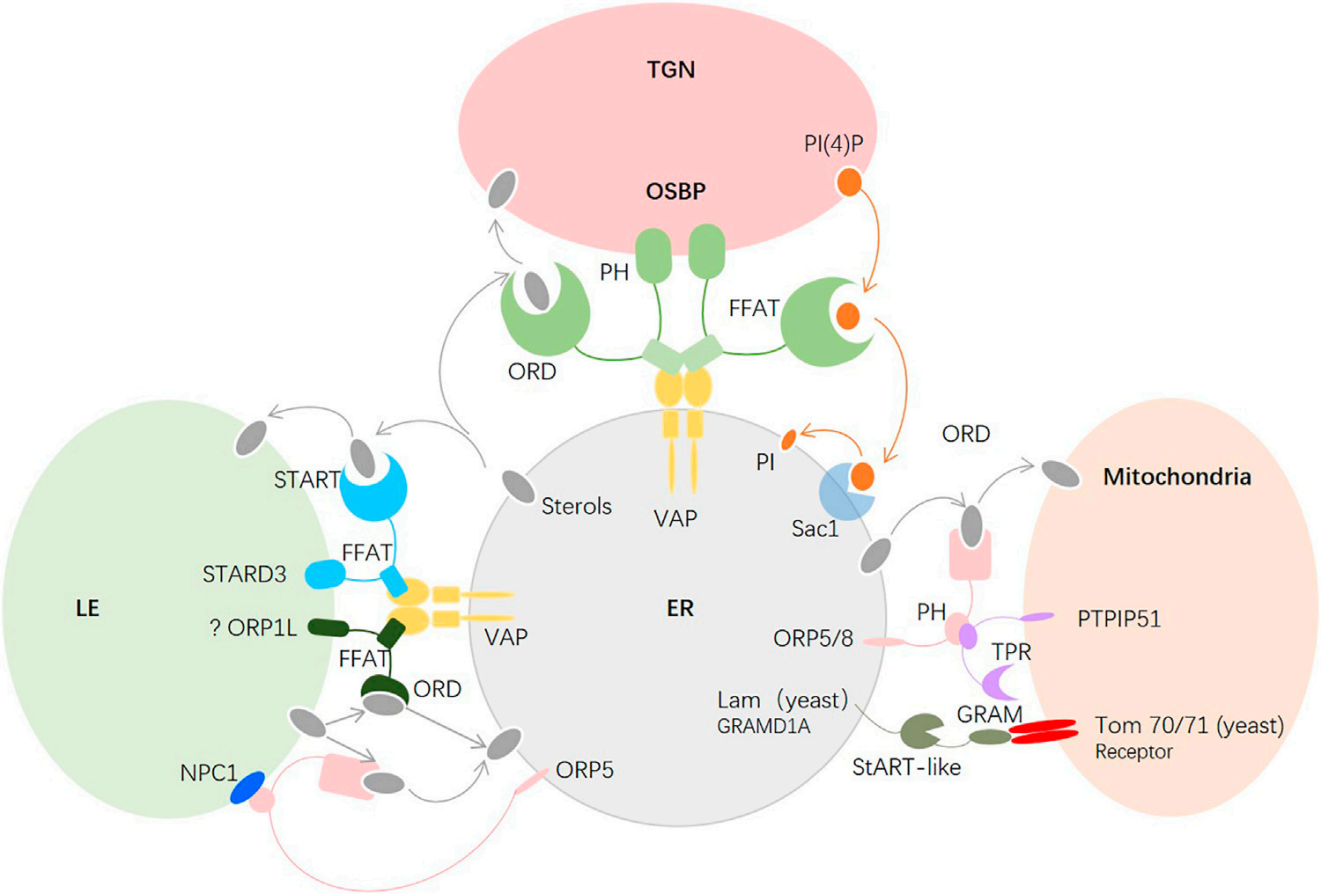
Major molecules in intracellular cholesterol transport. Between the ER and the TGN, OSBP bridges the two membranes, sterols of the ER that bind to the ORD are transferred to the TGN, and the ORD of OSBP transfers PI(4)P of the TGN back to the ER. Between the ER and the mitochondria, ORP5/ORP8 of the ER and PTPIP51 of the mitochondria tether the two organelles at the MAM, and the ORD of ORP5/8 transfers the sterols of the ER to the mitochondria. In yeast, Lam6p located in the ER facilitates the sterol transfer by interacting with Tom70/71 in the OMM. The conserved mammalian ortholog of Lam6p is GRAMD1A, which is proposed to interact with the receptor of the mitochondria to transfer sterols.

The contacts of the ER with the PM, mitochondria, endosomes, peroxisomes, Golgi, and lipid droplets are mediated by membrane contact sites (MCSs), which are membrane microdomains formed between two organelles close to each other (∼10–30 nm) (Wang and Dehesh, 2018; Martello et al., 2020). Many LTPs are localized to MCSs and undergo conformational changes from open bridges to closed tubes to facilitate the transfer of lipids (Figure 3). To date, at least 27 protein families have been found in lipid trafficking.

#### Regulation of Cholesterol Transport From the Endoplasmic Reticulum

Most newly synthesized cholesterol is transported to the trans-Golgi network (TGN), which is a sorting site for lipids, to maintain a low concentration in the ER. Oxysterol-binding protein (OSBP), a bridge between the ER and Golgi membranes, and has been observed to mediate cholesterol transfer. OSBP contains three conserved domains: the N-terminal pH domain, the central FFAT motif, and the C-terminal OSBP-related domain (ORD), which recognize PI(4)P and small GTPase ADP-ribosylation factor (Arf1) in the Golgi, target the VAP-A protein in the ER, and bind sterols, respectively. The architecture of OSBP supports cholesterol export (Antonny et al., 2018). In detail, first, the membranes are tethered between Golgi and ER by the pH domain and FFAT motif of OSBP; second, sterols that bind to the ORD are transferred to the Golgi; third, at the Golgi, the ORD of OSBP transfers PI(4)P, which is synthesized by phosphatidylinositol 4-kinase (PI4K) IIIβ, back to the ER; and fourth, PI(4)P is dephosphorylated to PI *via* Sac1, which is an ER-localized phosphatase. The low ratio of PI(4)P to sterols in the ER makes the phosphorylation and dephosphorylation cycle move continuously to fuel cholesterol export. The exchange between cholesterol in the ER and PI(4)P in the Golgi is maintained by PI4KIIIβ and Sac1 (Antonny et al., 2018). Intriguingly, Sac1 also acts in trans on 4-phosphatase on PI(4)P in a manner mediated by FAPP1 when the concentration of PI(4)P is elevated in the TGN (Venditti et al., 2019). The two modes of Sac1 activity may coexist in cells such that when the concentration of PI(4)P reaches a threshold, the trans-phosphatase activity of Sac1 is enhanced and coordinated with the in *cis* phosphatase activity to lower PI(4)P levels in the TGN. Moreover, the in *cis* activity of Sac1 is required for contact sites between the PM and the ER or the late endosomes (LEs) and the ER (Del Bel and Brill, 2018). A recent study found that in cholesterol-fed cells, the ER-anchored cholesterol escort SCAP interacts with the VAP-OSBP complex *via* Sac1. Deletion of SCAP inhibits PI(4)P transport and carriers of the Golgi network to the cell surface (CARTS) (Wakana et al., 2021). Whether cholesterol perturbation causes disruption of the cycle between PI(4)P and cholesterol is unclear.

Mitochondria are important organelles in cells that can synthesize phosphatidylglycerol, cardiolipin, and phosphatidylethanolamine but must import phosphatidylcholine, phosphatidylinositol, PtdSer, and sterols from other organelles to maintain normal function (Flis and Daum, 2013; Horvath and Daum, 2013). The inner mitochondrial membrane (IMM) has abundant proteins and only 20% lipids, while the outer mitochondrial membrane (OMM) is lipid rich in mammalian cells. The ER and mitochondria are physically connected at the mitochondria-associated membrane (MAM). Most cholesterol transfer from the ER to mitochondria takes place on the MCSs of MAMs (Giordano, 2018). There are three families of LTPs conserved in yeast and mammals as tethers, lipid sensors, or transporters at the MCSs between the ER and mitochondria. The first is the ORP family; specifically, ORP5 and ORP8 interact with tyrosine phosphatase-interacting protein 51 (PTPIP51) at the MCSs and mediate ER-mitochondrial contact as well as at the PM-ER to facilitate sterol transport in mammalian cells (Chung et al., 2015; Galmes et al., 2016). The second is the START family, which is responsible for cholesterol transport from the OMM to the IMM under hormonal stimulation, after which the cholesterol in the IMM is transformed into pregnenolone for production of steroids or bile acid in hepatic cells (Elustondo et al., 2017). The third is the LAM-GRAM family, which was recently discovered in yeast and includes Lam6 and Lct1, which are ER-anchored proteins located in the ER-mitochondria MCSs that bind with the mitochondrial import receptors Tom70 and Tom71 in yeast (Murley et al., 2015). The conserved orthologs in mammals are GRAMD1A and GRAMD1C, which are involved in lipid transfer in the PM (Naito et al., 2019; Ercan et al., 2021; Ikonen and Zhou, 2021), but their localization and function remain to be elucidated. Thus, we know little about cholesterol transfer at the ER-mitochondria MCSs in mammals at present. The discovery of new sterol transfer molecules will further illustrate the important roles of cholesterol and MCSs in mitochondria.

Endosomes also have abundant contact sites with the ER, and cholesterol is transferred from the ER to late endosomes (LEs) and lysosomes (LYs) *via* MCSs in cells. StAR-related lipid transfer protein 3 (STARD3), also known as MLN64, contains a conserved FFAT-like motif that interacts with VAPs in the ER membrane, mediates MCS formation between the ER and LE and transfers newly synthesized cholesterol from the ER to endosomes via a sterol-binding domain (Wilhelm et al., 2016). Similar to another sterol transfer protein, ORP1L, which responds to cholesterol transfer from endosomes to the ER, STARD3 binds VAP to form a tether between the ER and endosome (Ridgway and Zhao, 2018). Whether these proteins compete with each other for VAP binding and how the major molecule that binds with VAP is regulated needs further investigation.

#### Regulation of Cholesterol Transport From Late Endosomes/Lysosomes

Cholesteryl esters (CEs) carried by low-density lipoprotein (LDL) are absorbed by LDL receptors (LDLRs) at the membrane and hydrolyzed by acid lipase in LEs. The released free cholesterol is transferred to other organelles: ER, PM, mitochondria, TGN, and peroxisomes. This transfer of cholesterol from LEs/LYs is also mediated by sterol transfer proteins (STPs) at MCSs. ORP1L and ORP5 respond to cholesterol transfer from LEs/LYs to the ER (Ridgway and Zhao, 2018). Additionally, ORP5 is responsible for the cycling of PS in the ER and PI(4)P in the PM to maintain the low level of PI(4,5)P2 in the PM (Ghai et al., 2017). During the movement of cholesterol from LEs/LYs to mitochondria, STARD3 also plays an important role by accepting NPC2-bound LDL-C to directly bypass NPC1 and transfer the LDL-C to the mitochondrial membrane *in vitro* (Charman et al., 2010). Peroxisomes, as sites of lipid metabolism, play an important role in the cholesterol trafficking pathway. Synaptotagmin VII (Syt7) of lysosomes and PI(4, 5)P2 of peroxisomes is located at MCSs that form between the two organelles. Either Syt7 or PI(4, 5)P2 is essential to the formation of the MCSs and to cholesterol export from LYs (Chu et al., 2015). Syt7 has been reported to be a potential oncogenic target and to be involved in synaptic transmission as a calcium sensor (Turecek et al., 2017; Fu et al., 2021). Thus, further side effects need to be studied intensively when targeting Syt7 to cure disease.

## Discussion

Cholesterol is an essential lipid that serves as a precursor of steroid hormones, bile acids, and oxysterols in special mammalian tissues. Disturbed cholesterol homeostasis in humans is related to cardiovascular disease, cancer, neurodegenerative disease, and congenital disease. Thus, the *de novo* synthesis of cholesterol in cells and regulation, coordination between intracellular syntheses, import of exogenous cholesterol, biological distribution in organelles, transport of cholesterol in and out of cells, trafficking of intracellular cholesterol, and how to coordinate all the above processes precisely need to be researched continuously.

Because of the central role of SREBP2 in cholesterol homeostasis, numerous dysregulations of the gene in certain disease phenotypes are connected to cholesterol homeostasis. Some investigations have revealed that SREBP2 can function independently in addition to regulating cholesterol synthesis. For example, in circulating melanoma cells, SREBP2 contributes to ferroptosis resistance by inducing transcription of the ion carrier transferrin (TF) (Hong et al., 2021); in coronary artery endothelial cells, high-mobility group box 1 (HMGB1) attenuates LDL transcytosis by inhibiting SREBP2 (Ghaffari et al., 2021); and in idiopathic pulmonary fibrosis (IPF) patients, SREBP2 is markedly increased, and overexpressed SREBP2 in endothelial cells (ECs) enhances the TGF and Wnt pathways and mesenchymal genes *in vitro* and exacerbates vascular remodeling *in vivo* (Martin et al., 2021). Therefore, SREBP2, as a transcription factor, not only plays a key role in cholesterol homeostasis but also exerts multifunctional effects in pathophysiology. The additional functions and related mechanisms need further investigation.

The newly synthesized cholesterol and the released free cholesterol hydrolyzed from endocytosis LDL-C need to be distributed rapidly to maintain the normal functions of cells. Although more LTPs are identified and closely connect with MCSs in membranes, the detailed mechanisms by which they facilitate cholesterol transfer, and whether they have other pathophysiological roles and can be inhibited as drug targets, are still not well known.

For example, the well-known function of STARD3 is to tether the ER and endosome and facilitate cholesterol transfer from the ER to the endosome. Recent findings indicate that high STARD3 levels are associated with worse overall survival (OS), relapse-free survival (RFS), and disease metastasis-free survival (MFS). STARD3 expression is associated with HER2+ breast cancers (BCs); thus, STARD3 has the potential to be a diagnostic and predictive marker of HER2+ BC (Asif et al., 2021). Moreover, increased STARD1 expression is found in Alzheimer’s disease (AD) and Down syndrome (DS), and AD and DS patients exhibit lysosomal cholesterol accumulation within hippocampal astrocytes (Arenas et al., 2020). Thus, STARD1 could be a preclinical marker of AD at early stages. In alcoholic liver disease (ALD), STARD1 not only acts as a sterol transporter but also serves as a UPR and ER stress gene, which is stimulated by alcohol and facilitates ALD development (Marí et al., 2014). Moreover, STARD1 is expressed in many extra-adrenal and extra-gonadal organs, cells, and malignancies, including brain, eye, liver, vasculature, macrophages, heart, lung, skin cells, and so on. In liver, STARD1 involved in bile acid formation *via* the “alternative acidic” pathway, in which the translocated cholesterol in IMM is catalyzed into oxysterols, including 27,24,25-hydroxycholesterol, activated liver X receptors (LXRs) to facilitate bile acid production (Anuka et al., 2013). In addition, in macrophages, STARD1 also facilitated the cholesterol efflux by activate LXRs (Taylor et al., 2010; Manna et al., 2015). The functions of STARD1 in extra-endocrine tissues need more attention in future research.

The functional ORD of ORP5 interacts with mTOR1 and participates in cancer cell invasion and tumor progression. ORP5 depletion impairs mTOR localization to lysosomes, abolishes mTORC1 activity, and inhibits cell proliferation in HeLa cells (Du et al., 2018). The oncogenic gene KRAS is anchored on PM to maintain biological activity. The C-terminal of KRAS binds with specificity to PtdSer in the PM. Both ORP5 and ORP8 are responsible for exchanging PtdSer in the ER and phosphatidyl-4-phosphate in the PM. Depletion of ORP5 or ORP8 reduces PtdSer in the PM, causes KRAS mislocalization *in vitro*, and attenuates KRAS signaling *in vivo*; in addition, it reduces cell proliferation of KRAS-dependent cancer cells (Kattan et al., 2019).

GRAMD1A, which facilitates lipid transfer between the mitochondria and the ER, similar to ORP5, promotes HCC self-renewal, tumor growth, and resistance to chemotherapy. The effects of GRAMD1A are mediated by STAT5 (Fu et al., 2016). In addition, during autophagosome biogenesis, GRAMD1A is bound by autogramins on its StART domain, causing accumulation of GRAMD1A at the sites of autophagosome initiation (Laraia et al., 2019).

As indicated for the above-mentioned molecules, although alterations in both cholesterol and its related genes are observed in certain pathological conditions simultaneously, the exact functions of the molecules aside from cholesterol regulation need to be further investigated.

The most extensive application of lipid-lowering drugs in the clinic is antiatherogenic to reduce the morbidity and mortality of cardiovascular disease. Targets in the clinical and preclinical stages include HMGCR, proprotein convertase subtilisin/kexin type 9 (PCSK9), apolipoprotein B (Apo B), apolipoprotein C-III (Apo CIII), angiopoietin-like 3 (ANGPTL3), lipoprotein(a) (LPA), and Niemann-Pick C1-Like 1 (NPC1L1) (Table 2); among these, only statins for HMGCR inhibition are in the cholesterol synthesis pathway, while the others are associated with the assembly, transport and absorption of low-density lipoprotein cholesterol (LDL-C), and the inhibition of triglyceride synthesis.

**TABLE 2:** Targets in lipid homeostasis for cardiovascular disease

Target	Full name	Mechanisms
HMGCR inhibitor: Statins	3-hydroxy-3-methylglutaryl coenzyme A reductase (HMGCR)	Reduces cholesterol synthesis
NPC1L1 inhibitor: Ezetimibe	Niemann–Pick C1-like 1 (NPC1L1)	Reduces intestinal absorption
PPARα agonist: Fibrates pemafibrate	Peroxisome proliferator activated receptorα (PPARα)	Reduces triglyceride in Liver
Bile acid sequestering agents	Bile acids	Reduce acids reabsorption and cholestrol levels
PCSK9 inhibitor antibodies/ASOs	Proprotein convertase Subtilisin/Kexin type 9 (PCSK9)	Reduces LDLR and LDL-C endocytosis
Apo B inhibitor Mipomersen (ASO)	Apolipoprotein B (ApoB)	Inhibits Apo B synthesis, LDL-C assembly
ACLY inhibitor: Bempedoic acid SB-204990 ETC-1002	ATP-citrate lyase (ACLY)	Inhibits ACLY caused decrease Acetyl-CoA and inhibit cholestrol synthesis
MTP inhibitor: Lomitapide	Microsomal triglyceride transfer protein (MTP)	Reduces Apo B-containing lipoproteins
Apo A inhibitor: AMG 890 AKCEA-APO(a)-LRx (ASO) IONIS-APO(a)Rx (ASO)	Apolipoprotein(a) (Apo A)/lipoprotein(a) [Lp(a)]	Reduces Lp(a) levels
CETP inhibitor: Evacetrapib anacetrapib	Cholesteryl ester transfer protein (CETP)	Increases HDL level and decrease LDL-C level
Apo C-III inhibitor Volanesorsen (ASO) ISIS 304801 (ASO)	Apolipoprotein C-III (Apo C-III)	Decrease triglyceride levels
Apolipoprotein A-I mimetic peptides: 4F	Apolipoprotein A-I (Apo A-I)	Increases HDLs
ANGPTL3 inhibitor: BE-Angplt3 Gln135 (gene editing) IONIS-ANGPTL3-LRx (ASO) Evinacumab	Angiopoietin like-3 (ANGPTL3)	Increase lipoprotein lipase (LPL) and EL activity
ANGPTL4 inhibitor 14D12	Angiopoietin like-4 (ANGPTL4)	Increases LPL activity

Aside from cardiovascular disease, increasing evidence indicates that dysregulation of cholesterol homeostasis or some related genes correlates with cancers (Kopecka et al., 2020), neurodegenerative disease (Dai et al., 2021), fibrosis (Ioannou, 2016), and viral infection (Kočar et al., 2021). For example, cholesterol- and lipid-mediated innate immune memory induces COVID-19-related cytokine storms (Sohrabi et al., 2021), and decreased cholesterol synthesis of invariant natural killer T cells reduces IFN-*γ* production in the tumor microenvironment (Fu et al., 2020). In AD, AD brains retain significantly more cholesterol than age-matched nondementia control (ND) brains; the APP acts as a lipid-sensing peptide on cholesterol and forms MAMs in the ER, causing extracellular cholesterol internalization in the ER (Montesinos et al., 2020). In addition to the antiatherogenic drugs approved by the Food and Drug Administration (FDA), several molecules in the mevalonate pathway have emerged as promising drug targets for cancer and AD. For example, *SC4MOL* and *NSDHL* inactivation sensitizes tumor cells to EGFR inhibitors (Sukhanova et al., 2013), and *DHCR24* heterozygous knockout in mice reduces cholesterol levels without causing health problems (Horvat et al., 2011). Therefore, further genetic screening of drug targets in the mevalonate pathway and cholesterol homeostasis for cancers and neurodegenerative disease therapy or prevention are essential. Targeting the mevalonate pathway or cholesterol homeostasis combined with medicine used in the clinic may benefit disease therapy.

In recent years, additional traditional Chinese medicines have been observed to have cholesterol-lowering effects, including aloe-emodin (Su et al., 2020), apigenin (Wu et al., 2021b), Dingxin recipe IV (Zhang et al., 2021), and ZeXie decoction (Wu et al., 2021a). The mechanisms of some of these medicines involve SREBP2 transcription and maturation processes. Therefore, it is worth testing additional traditional Chinese medicines based on the present medicinal knowledge.

Along with the increasing understanding of cholesterol homeostasis, more regulator molecules have been identified to be involved in pathological conditions. Targeting of related molecules has been demonstrated to ameliorate certain symptoms; however, more research is needed to assess the side effects. Aside from cholesterol itself, intermediates of the mevalonate pathway, lipid transfer proteins, and metabolites of cholesterol all warrant further research.

## Glossary

ER: endoplasmic reticulum
PM: plasma membrane
GPI: glycosylphosphatidylinositol
HMG-CoA: 3-hydroxy-3-methylglutaryl CoA
IPP: isopentenyl pyrophosphate
FPP: farnesyl pyrophosphate
HMGCR: 3-hydroxy-3-methylglutaryl coenzyme A reductase
SM: squalene monooxygenase
DFT: farnesyl-diphosphate farnesyltransferase
LSS: lanosterol synthase
PP: pyrophosphate
SREBP2: sterol regulatory element-binding protein 2
S1P: site 1 protease
S2P: site 2 protease
SRE: sterol regulatory element
IRE: insulin response element
Insig: insulin-induced gene
SCAP: SREBP cleavage-activating protein
PCK1: phosphoenolpyruvate carboxykinase 1
HCC: hepatocellular carcinoma
Brg1: Brahma-related gene 1
POST1: partner of site-1 protease
ERAD: endoplasmic reticulum–associated degradation
RNF145: ring finger protein 145
ORP: OSBP-related protein
USP20: ubiquitin carboxyl-terminal hydrolase 20
VCP: valosin-containing protein
GGpp: isoprenoid geranylgeranyl pyrophosphate
UBIAD1: UbiA prenyltransferase domain-containing protein 1
MARCH: membrane-associated ring-CH-type finger
UBE2G2: ubiquitin-conjugating enzyme E2 G2
UBE2J2: ubiquitin-conjugating enzyme E2 J2
PAQR3: adipoQ receptor 3
LTPs: lipid transfer proteins
MCSs: membrane contact sites
OSBP: oxysterol-binding protein
ORD: OSBP-related domain: OSBP-related domain
Arf1: ADP-ribosylation factor
PI4K: phosphatidylinositol 4-kinase
PTPIP51: phosphatase-interacting protein 51
IMM: inner mitochondrial membrane
OMM: outer mitochondrial membrane
MAM: mitochondria-associated membrane
GRAMD1: GRAM domain-containing 1
LEs: late endosomes
LYs: lysosomes
STARD3: StAR-related lipid transfer protein
CEs: cholesteryl esters
LDLR: low-density lipoprotein receptor
STPs: sterol transfer proteins
Syt7: synaptotagmin VII
TF: transferrin
HMGB1: high-mobility group box 1
IPF: idiopathic pulmonary fibrosis
ECs: endothelial cells
OS: overall survival
RFS: relapse-free survival
MFS: metastasis-free survival
BCs: breast cancers
DS: Down syndrome
ALD: alcoholic liver disease
ORD: OSBP-related domain: OSBP-related domain
PC: phosphatidylcholine
PtdSer: phosphatidylserine
PI: phosphatidylinositol
PCSK9: proprotein convertase subtilisin/kexin type 9
Apo B: apolipoprotein B
Apo CIII: apolipoprotein C-III
ANGPTL3: angiopoietin-like 3
LPA: lipoprotein(a)
NPC1L1: Niemann-Pick C1-like 1
